# A Device for Automatically Measuring and Supervising the Critical Care Patient’S Urine Output

**DOI:** 10.3390/s100100934

**Published:** 2010-01-26

**Authors:** Abraham Otero, Francisco Palacios, Teodor Akinfiev, Roemi Fernández

**Affiliations:** 1 Department of Information and Communications Systems Engineering, University San Pablo CEU, Boadilla del Monte 28668 Madrid, Spain; 2 Critical Care Unit, University Hospital of Getafe, Getafe, Carretera Toledo KM 12.500, 28901 Madrid, Spain; E-Mail: palaciosfra@gva.es; 3 Automatic Control Department, IAI/CSIC-Industrial Automation Institute, Spanish Council for Scientific Research, La Poveda, Arganda del Rey, 28500 Madrid, Spain; E-Mails: teodor@iai.csic.es (T.A.); roemi@iai.csic.es (R.F.)

**Keywords:** biosensors, patient monitoring, urine output, intelligent alarms

## Abstract

Critical care units are equipped with commercial monitoring devices capable of sensing patients’ physiological parameters and supervising the achievement of the established therapeutic goals. This avoids human errors in this task and considerably decreases the workload of the healthcare staff. However, at present there still is a very relevant physiological parameter that is measured and supervised manually by the critical care units’ healthcare staff: urine output. This paper presents a patent-pending device capable of automatically recording and supervising the urine output of a critical care patient. A high precision scale is used to measure the weight of a commercial urine meter. On the scale’s pan there is a support frame made up of Bosch profiles that isolates the scale from force transmission from the patient’s bed, and guarantees that the urine flows properly through the urine meter input tube. The scale’s readings are sent to a PC via Bluetooth where an application supervises the achievement of the therapeutic goals. The device is currently undergoing tests at a research unit associated with the University Hospital of Getafe in Spain.

## 1. Introduction

Current critical care units are equipped with modern and sophisticated commercial monitoring devices that are capable of sensing most of the patient’s physiological parameters. Heart rate, blood pressure, blood levels of oxygen saturation, respiratory rate, brain waves, and intracranial pressure are just a few examples. They provide physicians with an electronic recording of physiological parameters that can be inspected at any time. Most of these monitoring devices also allow physicians to establish therapeutic goals for the parameters they record. The fulfillment of these therapeutic goals is automatically supervised and, if a violation occurs, the devices generate audible warnings to alert the health care staff [1, 2, 3].

The overall result is a considerable reduction in the workload of the healthcare staff, since they do not need to continuously supervise the physiological parameters of each of the patients of the critical care unit. Furthermore, as with any repetitive and monotonous task, supervision of the temporal evolution of the patient’s physiological parameters is prone to errors [2, 4]. Many of these errors are avoided when such tasks are automated.

Arguably, the most relevant physiological parameter that is still measured and supervised manually by critical care unit staff is urine output (UO). This parameter is the best real-time indicator of the state of the patient’s kidneys. If a kidney is producing an adequate amount of urine it means that it is well perfused and oxygenated. An inadequate amount indicates that the patient is affected by some pathology.

When the UO of a patient is too low, the patient is said to have oliguria. If the patient does not produce urine at all, then he/she is said to have anuria. The most common pathophysiologic mechanisms causing oliguria and anuria are (1) prerenal azotemia which can be the result of heart failure, infectious diseases and gastrointestinal diseases; (2) intrinsic kidney damage, which can be caused by acute tubular necrosis, rhabdomyolysis, medication and/or glomerulonephritis; and (3) postrenal azotemia by obstruction of the urine flow which can be caused by an enlarged prostate, compression of the urethra by a tumor, an expanding hematoma or fluid collection [5]. Sometimes, UO can be too high; in these cases the patient is said to have polyuria. Diabetes is widely recognized as the most common cause of polyuria [6].

Urine output is also essential for calculating the patient’s water balance; and is used in multiple therapeutic protocols to assess the reaction of the patient to the treatment. Two of the more prominent clinical algorithms where UO plays a central role are the resuscitation of septic shock patients [7], and the resuscitation and early management of burn patients [8]. In the latter, while the end points of resuscitation are debatable, hourly UO is a well-established parameter in the fluid management of these patients, as well as one of the most reliable assessments of the patient’s state and evolution. In the case of the septic shock patient resuscitation, achieving a certain minimum value for the UO itself is a therapeutic goal.

**Figure 1.**
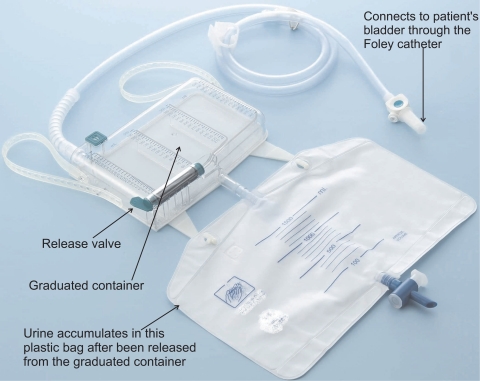
Commercial urine meter used in critical care units.

To measure the patient’s UO, a Foley catheter is introduced through the patient’s urethra until it reaches his/her bladder. The other end of the catheter is connected to a flexible plastic tube which is connected to a graduated container where the urine is collected. Every hour the nursing staff manually records the reading of the container of each patient, and operates a valve which releases the urine into a larger container (see Figure 1). Therefore, the healthcare staff does not benefit from the advantages of having a digital recording of this physiological parameter, or of the automatic supervision of its therapeutic goals. A device capable of automatically measuring the patients’ UO, and supervising the attainment of the established therapeutic goals, would release the healthcare staff from a considerable amount of work, and would permit measurements to be carried out more frequently.

This paper presents a patent-pending device [9] capable of recording and supervising the UO of critically ill patients. Section 2 explains the challenges of automating the sensing of UO, reviews briefly previous attempts to solve this problem, and describes the constraints that have guided the design of the sensor presented in this paper. Section 3 presents this sensor, the algorithms employed to supervise the therapeutic goals established for UO, and the results of a set of experiments that have allowed us to estimate the accuracy of the sensor’s measurements. Section 4 shows some preliminary results obtained with our device, and section 5 discusses this work. Finally, a series of conclusions and lines of future extension are given.

## 2. Automatic Sensing of Patient’s Urine Output

In the design of a sensor for measuring UO several considerations must be taken into account. On the one hand, any component of the sensor that is in contact, or may be in contact, with the urine of the patient cannot be reused for different patients. Furthermore, current commercial urinemeters usually need to be changed approximately every 4–7 days for hygienic reasons. Therefore, any component of the sensor that is or may be in contact with the urine must be easy to dispose of, and should have a low price.

Contact with urine also means that the component is in indirect contact with the patient’s bladder through the Foley catheter. Therefore, the components that are in contact or may enter into contact with the urine have to be sterilized before use.

Urine usually is acidic, although sometimes it can be slightly basic —its pH varies between 4.5 and 8. It usually contains uric acid (25–75 mg/L), urea (15–34 g/24 h), sodium (130–260 mEq/24 h), potassium (less than 90 mEq/24 h), chlorine (110–250 mEq/24 h), and Copper (less than 30 mcg/24 h), among other components [5]. Thus, it can be corrosive, especially for metals.

A sensor which aims to automatically measure UO must be able to provide feedback on urine production at least every hour, in order to generate similar information to that produced manually by the nursing staff. However, providing feedback at shorter intervals would be desirable. When a patient is producing a small amount of urine, it is more important to measure these quantities accurately. If the patient is producing a normal amount, the accuracy can be relaxed. Usually, the more urine a patient produces, the less relevant it is to have a highly accurate measurement.

Given the lack of sensors for measuring patients’ UO, there are no guidelines that specify the accuracy that they should provide, nor any consensus on what is an acceptable error when measuring this parameter. Before designing our sensor, we asked the medical team that collaborates with us to define, according to their criteria, what the accuracy requirements for this type of sensor should be. They provided the following requirements: The measurement error must not exceed

- 3 mL/h when the hourly UO is less than 40 mL/h
- 5 mL/h when the hourly UO is between 40 mL/h and 100 mL/h
- 10 mL/h when the hourly UO is between 100 mL/h and 250 mL/h
- 20 mL/h when the hourly UO is between 250 mL/h and 500 mL/h
- 40 mL/h when the hourly UO is over 500 mL/h.

Due to the lack of any other criteria, these are the ones we considered in the design of our sensor.

### 2.1. Previous Work

Ultrasound sensors have been proposed for use in the measure of the amount of liquid contained in a recipient [10]. However, the low accuracy of this type of sensor makes it unsuitable to measure volumes as small as those under consideration here. Although lasers have also been proposed for this task, their use is rather cumbersome because when a laser beam hits a liquid surface most of the beam power is transmitted. Float devices equipped with mirrors have been proposed to address this problem [11], but the final cost of the sensor makes it prohibitive for our application.

Commercial flowmeters are often used in industrial applications to measure the amount of fluid flowing through a tube. These devices are designed to measure a continuous flow of liquid, while UO is usually a small erratic drip. Furthermore, the sensor would have to be disposed of because it would be in direct contact with the urine, making it cost prohibitive.

To the best of our knowledge, only two devices have been proposed for automatically measuring patient’s UO. One of these devices, Urinfo 2000, was developed by the Israeli company Medynamix [12]. This device is based on a sensor that counts the number of drops of urine produced by the patient, and from this count estimates UO. The average error of the sensor when used to take hourly measurements was 8% (±25 mL). The authors provide no information on its performance when used to take more continuous measurements. Medynamix never marketed this device. At present, the company has been bought by Flowsense, a construction services company that seems to have discontinued Medynamix’s work on biosensors.

The second device was built by the authors of this article [13]. It is based on a sensor made up of two different volume containers. Both containers are connected, and they are arranged so that the larger container does not start filling until the smaller container is completely full. Both containers are equipped with a float which moves vertically along a pole. The floats allow us to detect when each container is full. A microcontroller detects the filling of the containers, releases their content by activating an actuator when it is required, and triggers an alarm if therapeutic goals are not being met.

The smaller container, which has a volume of 18 mL, is intended to provide an early warning of low UO. If the patient has oliguria, a precise and continuous monitoring of UO is needed. Thus, if the small container does not fill within the expected time, an alarm is triggered indicating a state of oliguria, and when the small container fills it will be emptied. When the patient is producing a very small amount of urine, the small container will be filled a few times a day; therefore, the actuator and the valve it opens and closes will not be subjected to significant stress. However, if the patient were producing normal amounts of urine or, even worse, if he/she had polyuria—these patients may produce up to 1 L of urine per hour—a container of such a low volume would require its content to be released up to several hundred times per day. Carrying out several hundred operations per day can cause considerable stress both on the release valve and on the actuator. The result would be an increase in price of an actuator and valve capable of supporting this stress without breaking the sterility of the device.

To solve this problem, when the patient does not have oliguria—and thus the small container fills within the expected time—the microcontroller does not releases the small sensor’s content when it gets full, but it waits until both containers are full. The sum of the volumes of both containers is approximately 180 mL. Therefore, for a patient who has severe polyuria, the actuator will be triggered no more than a few dozen times a day.

The device presented in [13] is capable of providing feedback on the patient’s UO with at least the same measuring interval used when manually supervising this parameter—1 hour—and in most cases within 20 or 30 min. However, when the intent is to use it in clinical studies that attempt to find correlations between UO patterns and the administration of drugs or the patients’ pathology evolution, this device has several drawbacks. On the one hand, the device only measures the instants of time at which urine production reaches certain values, but it provides no information on the rate of urine production between two consecutive measurements. On the other hand, it is not easy to build a sterile prototype of the custom sensor and actuator that are compliant with all laws applicable to a device that would be in indirect contact with the patient’s bladder.

### 2.2. Design Constraints for the New Device

When designing the device presented in this paper, we strived to design a device that would measure UO as accurately as possible, with the minimum acceptable accuracy provided by the requirements of our medical team. All components of the device that make contact with the urine would be easy to dispose of and cheap. The device would have to be built with readily available components, to simplify the prototype construction. Finally, it would be compliant with all the laws that apply to commercial urine meters, so the device could be approved by a Hospital Ethics Committee for the purpose of conducting clinical studies in critical care patients.

A simple solution to building a law compliant urine meter capable of automatically recording and supervising UO is to incorporate a commercial urine meter into the research device without breaking its sterility. We evaluated two different approaches to achieve this. The first one involved using a video camera to take pictures of the commercial urine meter in order to calculate the amount of urine produced from the images. The second approach required the use of a high-precision industrial scale. The first alternative requires greater computing power for processing the images, and relies on a careful positioning of the urine meter in order to function properly. The problem with the careful positioning of the device is aggravated by the fact that with this solution the healthcare staff would still have to open the graduated container valve every hour, and possibly modify the position of the device while doing so. Furthermore, a high precision scale can provide greater accuracy in the measurements. Thus, we opted for using the scale.

## 3. The device

### 3.1. Measuring Urine Output: The Sensor

To build our device we used the high-precision industrial scale PGW 4502e, built by Adam Equipment Inc. It has an accuracy of 0.01 g and a maximum capacity of 4500 g. We also used the commercial urine meter Unometer 500 built by Unomedical. The urine meter consists of two containers. One is a graduated plastic container with 500 mL of capacity which is equipped with a top opening with a filter that is used to equalize the internal and external pressures without risk of bacterial contamination. The second container is a flexible polymer plastic bag with 2000 mL of capacity. The containers are connected by a rigid tube and a valve, which in our device is always open; thus the two containers can be viewed as a single compartment. The Foley catheter connects to a polymer plastic, flexible, transparent, 110 cm length tube that is connected to the graduated container of the Unometer 500.

**Figure 2.**
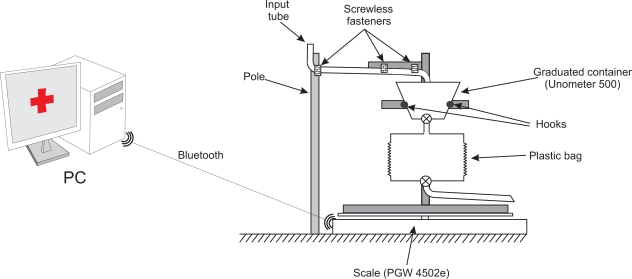
Device design.

We attached a metal structure built with Bosch profiles to the scale’s pan to hold the commercial urine meter (see Figure 2). The graduated container of the Unometer 500 is equipped with two hooks, one placed on each side of the container. We have used them to hang the device from the Bosch profile structure. Two screwless fasteners hold the input tube to the metal structure, and a third screwless fastener fixes the tube to a vertical pole. The purpose of the pole is to ensure that the input tube always lies in such a way that it does not hinder the flow of urine and it does not interfere with the operation of the scale. To this end, any portion of the tube between the pole and the Bosch profile structure must form a slightly negative angle with the horizon, to ensure that urine flows properly. However, at least in a portion of the tube, the angle must be small enough to prevent the transmission of forces from the bed of the patient towards the scale. An appropriate value for the angle formed by the tube and the horizon would be at least 5° at every point and, in at least a portion of the tube, the angle must be less than 10° to avoid force transmission.

**Figure 3.**
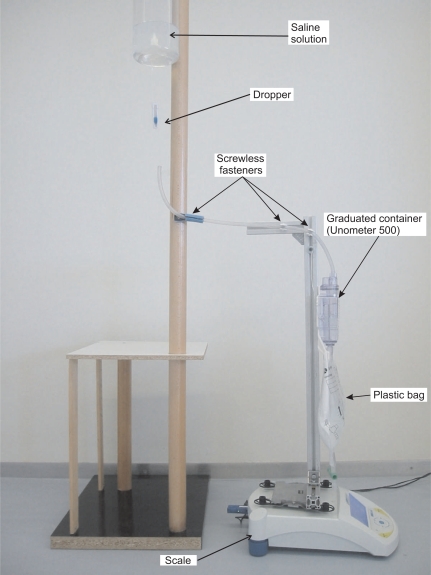
Picture of the urine meter prototype.

The PGW 4502e scale is provided with a RS232 port. By sending a command to this port it is possible to obtain the scale reading. To avoid the need for wiring the scale to the PC that takes the readings, a serial-port-to-Bluetooth adapter is used. This allows us to take readings from any PC equipped with a Bluetooth interface. A picture of our prototype device can be seen in Figure 3.

### 3.2. Processing the Measures

A Java application installed on a PC receives the readings through Bluetooth. The PGW 4502e scale can be configured to send measures only when the weight detected by the scale is stable. This feature is highly effective at removing transient artifacts, like those which may be produced by the nursing staff interacting with the commercial urine meter, or inadvertently touching the frame placed on top of the scale’s pan. The drawback of this feature is that the acquisition rate of scale measurements can vary: when this feature is enabled, the scale does not reply to the send measure command until the weight measured is stable.

Despite this disadvantage, we decided to use this feature because it is highly effective in removing artifacts. When the UO measure is not obtained at the expected time, cubic interpolation is used to generate a measure at the desired instant. In the cubic interpolation, we use the reading that was not taken at the desired instant, plus the three previous readings.

The acquisition sampling rate can be configured from the Java application itself, ranging from one minute to an hour. If the application waits more than five minutes for the response to the send measure command; *i.e.*, the weight detected by the scale is not stable for more than five minutes, the program generates an audible warning to alert the healthcare staff that the device is malfunctioning.

The Java application allows the healthcare staff to inspect a graph showing the patient’s UO in milliliters per hour. Furthermore, the therapeutic goals for UO can be established using this program. These therapeutic goals are represented with the aid of the Fuzzy Set Theory, a tool which has proved its value for handling and representing experience-based heuristic knowledge, such as that commonly used in the medical domain [14].

We shall introduce some basic concepts of the Fuzzy Set Theory which are the basis for our solution to supervise therapeutic goals. Given a discourse universe ℝ we define the concept of *fuzzy value A* by means of a possibility distribution *π^A^* defined over ℝ [15]. Given a precise value *x* ∈ ℝ, *π^A^*(*x*) ∈ [0, 1] represents the possibility of *A* being precisely *x*. A *fuzzy number* [16] is a normal and convex fuzzy value. A fuzzy value *A* is normal if and only if ∃*x* ∈ ℝ, *π^A^*(*x*) = 1. *A* is said to be convex if and only if ∀ *x, x*′, *x*″ ∈ ℝ, *x*′ ∈ [*x, x*″], *π^A^*(*x*′) ≥ *min*{*π^A^*(*x*), *π^A^*(*x*″)}.

We obtain a fuzzy number *A* from a flexible constraint given by a possibility distribution *π^A^*, which defines a mapping from ℝ to the real interval [0, 1]. A fuzzy constraint can be induced by an item of information such as *“x has a high value”*, where *“high value”* will be represented by *π*^*A*=*high*^. Given a precise number *x* ∈ ℝ, *π*^*A*=*high*^(*x*) ∈ [0, 1] represents the possibility of *A* being precisely *x; i.e.*, the degree with which *x* fulfills the constraint induced by *“high value”*.

**Figure 4.**
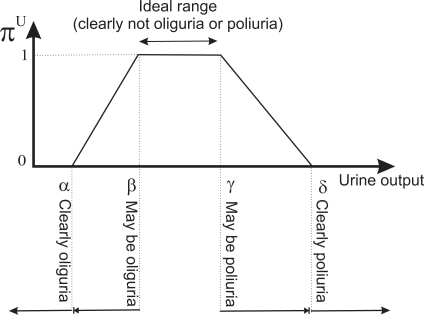
Trapezoidal possibility distribution representing the therapeutic goal for urine output.

Normality and convexity properties are satisfied by representing *π^A^*, for example, by means of a trapezoidal representation. In this way, *A* = (*α*, *β*, *γ*, *δ*), *α* ≤ *β*, ≤ *γ* ≤ *δ*, where [*β*, *γ*] represents the core, *core*(*A*) = {*x* ∈ ℝ| *π^A^*(*x*) = 1}, and ]*α, δ*[ represents the support, *supp*(*A*) = {*x* ∈ ℝ|*π^A^*(*x*) > 0} (see Figure 4). We have opted for the trapezoidal representation owing to its computational efficiency and the intuitiveness of its semantics. Furthermore in possibility theory, rather than the precise assignment of possibility degrees, what matters is the order of the possibility degrees attached to different values.

We shall represent the therapeutic goals for UO by the trapezoidal possibility distribution *π^U^*. The beginning of the support of the distribution is a value such that if the patient is producing that amount of urine, or less, the patient clearly has oliguria. The beginning of the core is a value such that if the patient is producing that amount of urine, or more, the patient clearly does not have oliguria. The end of the core is a value such that if the patient is producing that amount of urine, or less, the patient clearly does not have polyuria. The end of the support is a value such that if the patient is producing that amount of urine, or more, the patient clearly has polyuria. The beginning and end of the core of the distribution limit the interval within which ideal values of UO lie; *i.e.*, the interval where the patient clearly has neither oliguria nor polyuria (see Figure 4).

Given the definition we use for the beginning and end of the support and core of the distribution, the acquisition of the supervision criteria is simple and easily understood by the healthcare staff. On the other hand, by representing therapeutic goals with a possibility distribution we avoid errors produced by employing some arbitrary crisp limits when classifying the state of the patient in “oliguria”, “ideal state” and “polyuria”.

**Figure 5.**
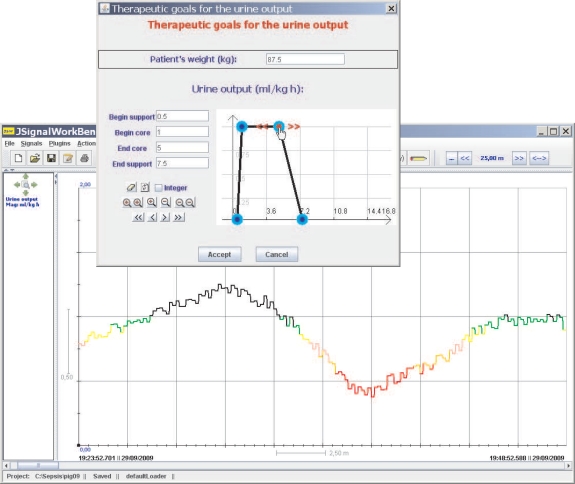
Main screen of the Java application showing urine output and the window that allows healthcare staff to set therapeutic goals.

Physicians are accustomed to expressing the therapeutic goals for UO in milliliters per kilogram of patient body mass per hour (*mL/kg · h*). Our tool allows them to specify the therapeutic goals—*π^U^*—in these units. In order to be able to convert the information provided by the scale to *mL/kg · h*, we also need physicians to indicate the weight of the patient—*P* —(see Figure 5). Let us suppose that the reading provided by the scale at the instant *t*_*i*−1_, *i* ≥ 1, is *W*_*i*−1_; and that the following reading, taken at *t_i_*, is *W_i_*. The density of urine is between 1.005 and 1.035 g/mL, and has an average value of approximately 1.020 g/mL. Therefore, the amount of urine produced during the interval [*t_i_*, *t*_*i*+1_], measured in *mL/kg · h*., will be:

(1) $$u_{i}=\frac{W_{i}-W_{i-1}}{(t_{i}-t_{i-1})\cdot 1.020\cdot P}$$

where *P* is the patient’s weight in kilograms, *W_i_* − *W*_*i*−1_ must be expressed in g, and *t_i_* − *t*_*i*−1_ must be expressed in hours.

*π^U^* can be interpreted as a computational projection of the piece of clinical knowledge “adequate UO”. Thus, the degree to which the therapeutic goals established for the patient are being met is given by *π^U^* (*u_i_*). If *π^U^* (*u_i_*) = 0, either UO is less than the minimum acceptable value—the patient clearly has oliguria or anuria, or it is greater than the maximum acceptable—the patient clearly has polyuria. If *π^U^* (*u_i_*) = 1, UO is within the range of ideal values. The closer *π^U^* (*u_i_*) is to 1, the closer the amount of urine produced by the patient is to the ideal value, and the closer *π^U^* (*u_i_*) is to 0, the closer the patient is to oliguria or polyuria.

*π^U^* (*u_i_*) is useful information in assessing the patient’s state. However, a possibility degree between 0 and 1 is not the best representation of information that has to be presented to healthcare staff. A color code used when drawing the graph of UO is a much more suitable solution, and it has been used successfully by the authors on similar problems [1, 17]. Red represents the null compatibility—the patient clearly has oliguria or polyuria–, followed by orange, pink, yellow, green and black, which represents the total compatibility—the UO lies within the range of ideal values. In this way, the UO graph provides instantaneous visual feedback on the patient’s state (see Figure 5).

Besides providing visual feedback, a monitoring device should have audible warnings to alert the healthcare staff about serious deviations from therapeutic goals. The application produces an audible warning until it is turned off by the healthcare staff whenever *π^U^* (*u_i_*) = 0. It also produces a different audible warning when the scale does not respond to the send measure command within five minutes to warn of a malfunction in the device. Finally, the program can be configured to emit a third type of audible warning when the total urine production of the patient reaches a certain threshold. The purpose of this alarm is to alert healthcare staff when it is time to empty the plastic bag of the commercial urine meter. Before emptying the bag, the healthcare staff must click a button to indicate that this action is going to be performed. When the button is clicked, the program takes one last measure from the scale, stops monitoring, and informs the healthcare staff that the bag can be emptied. Once this process has been completed, the button must be clicked again to resume monitoring.

### 3.3. Accuracy of the Sensor

The main source of error in the measures is the approximate value used for urine density in Equation 1. This value varies between 1.005 and 1.035 g/mL; thus by using the average value of 1.020 g/mL the maximum error in the measurements will be approximately 1.5%. The Java application allows healthcare staff to modify the urine density value if the exact value is known, although this is seldom the case.

The high-precision industrial scale we used has a maximum error ensured by the manufacturer of ±0.01 g. Thus, this error is negligible when compared to the error in estimating urine density. However, this error can only be applied to measurements in which an object is placed on the scales’ pan and the measure is immediately taken. When the scale is used to record continuous measurements over time, as in our application, room temperature variations can affect the scale and produce an additional source of noise.

**Figure 6.**
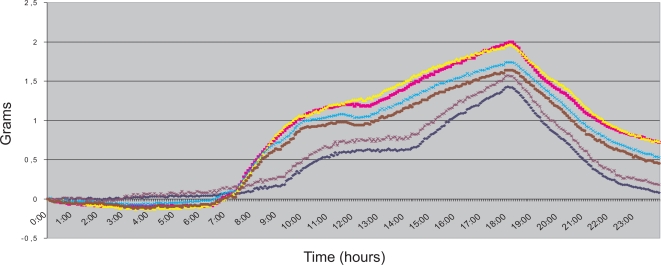
Noise caused by variations in room temperature

To characterize this noise we performed several tests where the readings of the scale were recorded over 6 days, taking one reading every five minutes. The weight placed on the scale’s pan remained constant throughout each day. Therefore, deviations from the initial weight correspond to errors in the measure. Figure 6 shows the scale readings taken during the six days. They are represented as six different time series, each one corresponding with the readings taken from 00:00:00 to 23:59:59 of every day. All measures started at 00:00:00; thus the value of all the time series in this instant is 0 g.

The public building where the measurements took place opens its doors at 07:00. Air conditioning is switched on at 07:30. The air conditioning is switched off at 18:00. The operation of air conditioning, in all probability, causes the increase in scale readings between these hours. The continued decrease that begins shortly after 18:00 very likely corresponds with the return to the initial state after the air conditioning has been switched off. During night hours, readings are quite stable.

In a critical care unit, room temperature is controlled by air conditioning 24 hours a day, usually remaining between 20° and 24° C. During our experiments, the variations in room temperature were up to 10° C. Therefore, we can conclude that, for our application, the situation shown in Figure 6 corresponds with a worst case scenario of room temperature variation. In our tests, the maximum hourly variation on the scale readings was slightly over 0.3 g. This leads us to estimate that the maximum error produced by variations in room temperature when the device is employed in a critical care unit will always be within ±0.4 g ≃ ±0.4 mL per hour.

A third potential source of noise in the measures is caused by the characteristics of our experimental setup. A section of the plastic input tube of the commercial urine meter hangs between the frame located on top of the scale and the pole (see Figure 2). When this tube is placed by the healthcare staff, it will not be in a stable state, but will slowly change its position until a stable state is reached. The changes in the tube position are imperceptible to the human eye and they are subtle enough to be ignored by the stability filter of the scale, but they do influence the values registered by the scale.

A flexible tube hanging between two fixed points at the mercy of gravity changes its position at an exponentially decreasing rate until it reaches stability. The longer the tube section located between two fixed points is, the faster the tube reaches its stable state. The tube’s movement can cause more of the tube’s weight to fall over the scale or over the pole (see Figure 2). In the first case, the scale should register a weight increase, while in the second it should register a weight decrease.

**Figure 7.**
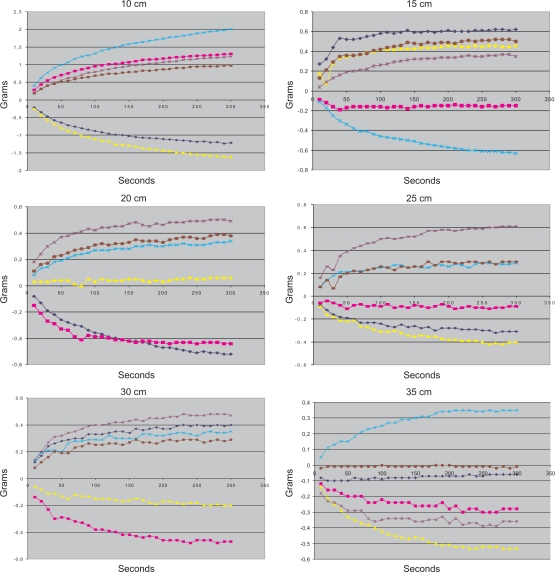
Behavior of the device during the five minutes just after the commercial urine meter was set up. Different tube lengths are shown.

To characterize this error we carried out a series of experiments in which we began to record the scale’s measurements just after the commercial urine meter was set up. The measures were taken every 10 seconds, for a total of five minutes. Experiments were conducted for different lengths of the section of the tube located between the frame placed on top of the scale and the pole. For each length, six different experiments were carried out. Results are shown in Figure 7.

For a section length of 10 cm, the error during the first five minutes can reach ±2 g, but as the length of the section of the tube increases, the magnitude of the errors decreases. For a section length of 35 cm, the error during the first five minutes remains within ±0.4 g, We can also see how, for every section length, the error of each new reading decreases exponentially. For all section lengths, except for 10 cm, the tube is stable after five minutes. Most of the drop of the tube occurs during the first minute, and after the first 150 seconds the tube can be considered to be in a stable state, except for the 10 cm tests.

Based on these experiments, the following guidelines for using the device were developed. The minimum length of the hanging tube section must be 20 cm, although longer lengths should be used if possible. At least two and half minutes must pass from the set up of the commercial urine meter until the time at which the recording is started. Under these conditions, we estimate that the error that the movement of the tube may cause during the first five minutes of recording should be under ±0.2 g ≃ ±0.2 mL.

Waiting at least two and half minutes before the start of the monitoring should not be a major problem given that before the monitoring can start, it is necessary to perform the urethral catheterization. This process requires approximately five minutes. If the healthcare staff set ups our device just before starting the catheterization, the tube will be stable when they have finished the procedure.

In summary, we estimate that the maximum error of our device when measuring hourly UO is ±1.5% of the patient’s UO, plus ±0.4 mL caused by the room temperature variation. For the first hour of monitoring, we must add ±0.2 mL caused by the input tube movement. The patient that produces just under 40 mL of urine during the first hour of monitoring provides the worst case scenario according to the constraints imposed by our medical team. In this case, the maximum error of our device is ±(1.5%·40 mL + 0.4 mL + 0.2 mL) ≃ ±1.2 mL. The maximum error that our medical team specified for this scenario was ±3 mL.

## 4. Results

A series of tests to verify the proper operation of the prototype device have been performed. In the tests a saline solution with similar properties to urine was used. This liquid was stored in a container placed at a higher level than the sensor, and a dropper was used to regulate the flow of fluid from this container to the sensor (see Figure 3). In the tests the amount of fluid sent to the sensor was carefully measured with a graduated cylinder in order to verify that the amount of urine detected by the Java application was accurate.

A stress test in which the system worked continuously for seven days was also conducted. After the seven days, it was found that both the scale as well as the Java application were still operating correctly and that the amount of urine detected by the application was correct.

After this initial validation, the device is been used in a research unit associated with the University Hospital of Getafe in Madrid, Spain. In this research unit, a series of experiments aimed at the study of sepsis in an animal model (pigs) are being conducted. Their goal is to gain a better understanding of the pathophysiological mechanisms underlying systemic and renal hemodynamics during sepsis. Sepsis is a serious medical condition characterized by a systemic inflammatory state that has developed in response to an infection and affects the patient’s entire body. Patients affected by this pathology are treated in an intensive care unit, and often require artificial ventilation and dialysis to support the functioning of the lungs and kidneys, respectively [18].

In these experiments, sepsis is induced by the administration of Escherichia coli –E. coli– bacteria. Animals are anesthetized and mechanically ventilated via tracheostomy. The following parameters are monitored invasively during the experiment: blood pressure, pulmonary arterial pressure (measured by a Swan-Ganz catheter), renal artery flow (measured by a Doppler sensor) and central flow (measured by a laser-Doppler probe). UO is continuously monitored by our device.

The fact that one of the goals of these experiments is to study renal hemodynamics makes the scenario ideal for testing our device [19]. Furthermore, these experiments allow us to refine our device. For example, after operating the device, physicians saw a need for the ability to place annotations over the temporal axis on the graph of UO. This would simplify the task of relating UO patterns with external events—represented by the annotations—such as the supply of a drug, the supply of a volume overload or any other therapeutic action. The feature has already been added to the Java application. If the initial animal trial is successful, before the end of this year we will start using our device in clinical studies with human patients admitted to the burn unit of the University Hospital of Getafe.

## 5. Discussion

The monitoring interval currently employed for UO—once every hour—establishes a compromise between avoiding risk states for the patient and not placing an excessive burden on the nursing staff. An automatic system, as the one described in this article, decreases the workload associated with monitoring UO and permits supervision to be carried out on a virtually continuous basis. Furthermore, the automation of the supervision of this parameter has the additional benefit of preventing human errors in this task.

To the best of our knowledge, no clinical studies have been conducted about the patterns of UO production where the monitoring interval was less than one hour. It may be the case that by carrying out the supervision of UO on a more continuous basis, healthcare staff can identify deviations from therapeutic goals at earlier stages, allowing the patient’s therapy to be modified more promptly, leading him/her more quickly to the desired state. A device for measuring UO on a virtually continuous basis with high accuracy, as the one presented in this article, is ideal for trying to find subtle correlations between UO patterns and drug therapy and/or the evolution of the patients’ pathology. Thus, it is ideal for carrying out studies where UO measures are taken more often than at present in critical care units.

Such clinical studies will also help define the optimum monitoring interval for UO, and determine the level of accuracy required for this task. In all likelihood, the accuracy of the device presented here is higher than required, and it is probably not necessary to monitor this parameter as continuously as our device permits. While this does not hinder the realization of clinical studies, it is a disadvantage for a commercial device designed to automate the monitoring and supervision of UO. In this situation, more accuracy than necessary and a higher acquisition rate than required would needlessly increase the price of the commercial device.

Currently, every hour a nurse must visit each of the patients’s beds of the critical care unit to manually record UO, must operate the valve that releases urine from the graduated container to the plastic bag, and must check if the plastic bag needs to be emptied. These tasks must be performed for each patient admitted to the critical care unit 24 times a day, 365 days a year. The solution presented in this paper completely eliminates these tasks, given that it not only automates the monitoring of UO, but it also can alert the nursing staff when it is necessary to empty the plastic bag. We estimate that all the tasks associated with UO monitoring for every 15 critical care unit patients during 24 hours require a time equivalent to one nursés workshift. Thus, even a partial automation of these tasks can yield economic savings for the institutions that provide healthcare services.

The maximum hourly measurement error of our device is ±1.5% of the patient’s UO during the hour (urine density variations) plus ±0.4 mL (room temperature variations). Currently, the measurement of urine is carried out visually. In the Unometer 500, for example, separations between the coarser measurement divisions in the 500 mL graduated container correspond with 20 mL; *i.e.*, a 4% error. However, this highly optimistic estimation of the error assumes that the visual measurements are taken in ideal conditions; *i.e.*, the graduated container is not tilted in any direction and the nurse’s eyes are in the same horizontal plane as the graduated container. In [12], a study on the accuracy of visual measurements of UO was performed. According to this study, in practice these errors are about 26%. In critical care units measures are not taken under ideal conditions. Besides this, two additional factors contributed to this high error. Sometimes the nurses did not properly close the graduated container valve; thus part of the urine produced during one hour leaked into the plastic bag and was not measured. Then, some patients had polyuria and they produced more than 500 mL of urine per hour. When this happens, the urine overflows from the graduated container and falls directly into the plastic bag, without being measured. The occurrence of these types of errors in critical care units increases the value of a device that automates the monitoring of UO.

Compared with the device the authors previously developed for monitoring UO [13], the one presented in this work has higher precision and is capable of measuring UO continuously, while the previous device was only capable of measuring the time that the patient required to produce certain amounts of urine. Furthermore, the process of building a sterile version of the device that can be used in tests with humans is much less complicated.

The device developed by Medynamix [12] automates the recording of UO, but it cannot supervise any therapeutic goals. Its readings are not transmitted to a PC, and it does not alert the health care staff when it is necessary to empty the urine meter’s plastic bag. Therefore, in practice the healthcare staff would need to check each patient every hour. According to its designers, the mean error of the device when used to measure hourly UO is 8%. This error does not meet the requirements specified by our medical team. In defense of Medynamix’s device, these criteria originate from an informed opinion of an experienced medical team rather than a consensus. Furthermore, when they provided these criteria, they had in mind a device aimed at conducting clinical studies about subtle UO production patterns, and not a device that it is just intended to automate the hourly monitoring of UO, as is the case of Medynamix’s device. It may well be that the accuracy of their device is indeed enough to properly measure hourly UO. However, this lower accuracy does make their device less appropriate than ours to conduct clinical studies.

## 6. Conclusions and Future Work

We have built a device capable of automatically monitoring and supervising the urine output of critical care patients. It is based on an industrial scale that provides a high-precision measurement of the weight of a commercial urine meter. To ensure the proper functioning of the urine meter and the scale, we have designed a frame that prevents force transmission from the patient’s bed and ensures that the urine flows properly. Scale readings are transmitted via Bluetooth to a Java application that runs on a PC. This application displays the patient’s urine output throughout his/her stay in the critical care unit, and alerts the healthcare staff of any deviation that occurs with respect to the established therapeutic goals.

Therapeutic goals are represented by a trapezoidal possibility distribution. The use of fuzzy logic for this task prevents mistakes when triggering alarms for patients which lie on the borderline between the ideal state and a pathological state—oliuguria or polyuria. When displaying the patient’s urine output, we use a color code that represents different degrees of deviation from therapeutic goals. Thus, our application provides an instantaneous visual feedback about the status of the patient’s urine output.

The main source of error in our device is produced by using an average urine density value of 1.020 g/mL to transform the scale measures (grams) to milliliters. This introduces an error of up to 1.5% in the measurements. Other sources of error are changes in room temperature, which affect the scale readings, and the initial transient state during which the position of the input tube of the commercial urine meter varies slightly. To characterize these errors, we have conducted a series of experiments. As a result, under normal operating conditions in a critical care unit, the maximum error in the measures is ±1.5% of the patient’s UO (urine density variations) plus ±0.4 mL (room temperature variations), plus ±0.2 mL for the first hour (tube movements). This error is considerably smaller than the error committed when taking visual measurements, and those committed by other devices proposed to measure urine output automatically [12, 13].

Currently, our device is being used in tests performed on animals in a research unit associated with the University Hospital of Getafe. As future work, we intend to use our device in the burn unit of this hospital, where we expect to conduct a series of clinical studies based on a more continuous and accurate monitoring of urine output throughout the stay of patients in a burn unit. The aim of these studies is to find relationships between urine output patterns and therapeutic actions, such as the administration of drugs, or the patients’ evolution throughout his/her pathology. The high accuracy and acquisition rate of our device make it ideal for carrying out such studies.
